# Self-Assembly of Low-Molecular-Weight Asymmetric Linear Triblock Terpolymers: How Low Can We Go?

**DOI:** 10.3390/molecules25235527

**Published:** 2020-11-25

**Authors:** Christina Miskaki, Ioannis Moutsios, Gkreti-Maria Manesi, Konstantinos Artopoiadis, Cheng-Yen Chang, Egor A. Bersenev, Dimitrios Moschovas, Dimitri A. Ivanov, Rong-Ming Ho, Apostolos Avgeropoulos

**Affiliations:** 1 Department of Materials Science Engineering, University of Ioannina, University Campus-Dourouti, 45110 Ioannina, Greece; xris.misk@gmail.com (C.M.); imoutsios@uoi.gr (I.M.); gretimanesi@uoi.gr (G.-M.M.); kartopoiades@gmail.com (K.A.); dmoschov@uoi.gr (D.M.); 2 Department of Chemical Engineering, National TsingHua University, Hsinchu 30013, Taiwan; joe950427@gmail.com (C.-Y.C.); rmho@mx.nthu.edu.tw (R.-M.H.); 3 Faculty of Chemistry, Lomonosov Moscow State University (MSU), GSP-1, 1-3 Leninskiye Gory, 119991 Moscow, Russia; bersenev.ea@phystech.edu (E.A.B.); dimitri.ivanov@uha.fr (D.A.I.); 4 Institute of Problems of Chemical Physics, Russian Academy of Sciences, Chernogolovka, 142432 Moscow, Russia; 5 Institut de Sciences des Matériaux de Mulhouse–IS2M, CNRS UMR7361, 15 Jean Starcky, 68057 Mulhouse, France

**Keywords:** linear triblock terpolymers, anionic polymerization, sequential addition of monomers, SEC, ^1^H-NMR, TEM, SAXS, self-assembly in bulk, Flory–Huggins interaction parameters (*χ*)

## Abstract

The synthesis of two (2) novel triblock terpolymers of the ABC type and one (1) of the BAC type, where A, B and C are chemically different segments, such as polystyrene (PS), poly(butadiene) (PB_1,4_) and poly(dimethylsiloxane) (PDMS), is reported; moreover, their corresponding molecular and bulk characterizations were performed. Very low dimensions are evident from the characterization in bulk from transmission electron microscopy studies, verified by small-angle X-ray data, since sub-16 nm domains are evident in all three cases. The self-assembly results justify the assumptions that the high Flory–Huggins parameter, *χ*, even in low molecular weights, leads to significantly well-ordered structures, despite the complexity of the systems studied. Furthermore, it is the first time that a structure/properties relationship was studied for such systems in bulk, potentially leading to prominent applications in nanotechnology and nanopatterning, for as low as sub-10 nm thin-film manipulations.

## 1. Introduction

It is well-known that microphase separation in block copolymers strongly depends on *χΝ* and *φ* values, where *Ν* is the degree of polymerization, *χ* is the Flory–Huggins interaction parameter and *φ* is the volume fraction of each block. Over the last decades, several two-phase morphologies have been obtained from numerous types of diblock copolymers, rendering their use in nanotechnology applications, due to the exquisite properties they exhibit, by altering their molecular characteristics (especially molecular weight and volume fraction) [1,2,3,4]. These copolymers can be applied as nanolithography masks [5,6,7,8,9], thin films [10,11,12,13,14], nanostructured membranes [15,16,17], etc. The use of various diblock copolymers in such applications has been extensively studied, but the ever-growing demand on novel morphologies, unattainable by diblock copolymers, has shifted the interest to corresponding linear triblock terpolymers [18].

Linear triblock terpolymers have been reported to exhibit well-defined three-phase morphologies, due to the third chemically different block. It is documented that the morphologies are significantly affected by the block sequence (ABC vs. BCA vs. CAB), the volume fraction ratio and the values of the Flory–Huggins interaction parameters (*χ*_AΒ_, *χ*_AC_ and *χ*_BC_) [19,20,21,22].

Table 1 summarizes the data on already reported linear triblock terpolymers in the literature [23,24,25,26,27,28,29,30,31,32,33,34,35,36,37,38,39,40,41,42,43,44,45,46,47,48,49,50,51,52,53,54,55,56,57,58,59,60,61,62,63,64,65,66,67,68,69,70,71,72,73,74], and, more specifically, block sequence, number (or weight) average molecular weight range and the annealing conditions used, together with the corresponding reference. It is straightforward that all cases already studied involve various types of block sequences with relatively high number average molecular weights, and their microphase separation is studied mostly after thermal annealing and appropriate staining conditions with only a few discrepancies evident.

In this study, we report the synthesis and the molecular and morphological characterization of two samples of the PS-*b*-PB_1,4_-*b*-PDMS type and one sample of the PB_1,4_-*b*-PS-*b*-PDMS sequence, exhibiting low values of total number average molecular weight, varying from 11.000 g/mol up to 14.000 g/mol. The aim of this work is twofold: (a) to examine the self-assembly capability in such systems and (b) whether or not the microphase separation is affected when the elastomeric PB_1,4_ block is either the first block or the middle block, respectively, despite the very low number average molecular weight of this specific segment in all samples. It is the very first time in the literature, to the best of our knowledge, that such low number average molecular weight triblock terpolymers were synthesized and their structure/properties relationship was studied.

## 2. Materials and Methods

Anionic polymerization under high vacuum techniques, through monomer sequential addition, was employed to prepare these samples, in order to be considered well-defined materials with low dispersity indices and exhibiting molecular/compositional homogeneity. The purification of all reagents (monomers and solvents), as well as the dilution of the initiator, is described extensively elsewhere [45,51,75,76]. The synthesis procedure for all three samples, together with the appropriate molecular characterization (number average molecular weights and mass fractions of the three blocks) via size exclusion chromatography (SEC; Supplementary Materials Figures S1–S3), vapor pressure osmometry (VPO) and proton nuclear magnetic resonance spectroscopy (^1^H-NMR; Supplementary Materials Figures S4–S6 and Table S1), is given in the Supplementary Materials. The synthetic steps for the two different block sequences (PS-*b*-PB_1,4_-*b*-PDMS and PB_1,4_-*b*-PS-*b*-PDMS, respectively) are illustrated in Supplementary Materials Scheme S1a,b. The instruments used for the molecular characterization, size exclusion chromatography or SEC (Agilent Technologies/Polymer Labs, St. Clara, CA, USA) and proton nuclear magnetic resonance spectroscopy or ^1^H-NMR (Bruker GmbH, Berlin, Germany) of the precursors, intermediated diblocks and final terpolymers are thoroughly described elsewhere [77]. The vapor pressure osmometry or VPO measurements were carried out at 45 °C, using dried toluene as solvent in a Gonotec 070 (Gonotec GmbH, Berlin, Germany) vapor pressure osmometer.

Differential scanning calorimetry (DSC) experiments, for the thermal characterization, were accomplished by employing a TA Instruments Q20 DSC (TA Instruments Ltd., Leatherhead, UK). Two heating cycles and one cooling cycle were made. The first heating concluded to the erasure of the thermal behavior of the sample. The heating and cooling rate in all cycles was 10 K/min. The thermographs given (Supplementary Materials Figures S7–S9) correspond to the second heating cycle.

High-resolution transmission electron microscopy (HR-TEM) measurements, for the self-assembly characterization, were carried out in a JEOL 2100 TEM (JEOL Ltd., Tokyo, Japan), by using 200 KeV as the acceleration voltage. The studied sample sections with thickness ~ 30 nm were placed on 600 mesh Cu grids and were taken through cryo-ultramicrotoming (Leica EM UC7 from Leica Microsystems, Wetzlar, Germany) at −140 °C (temperature well below the lowest T_g_ of the PDMS segments ~ −120 °C). The studied sections were examined before and after staining with vapors of OsO_4_ (2% *w*/*v* OsO_4_ in water (Science Services, Munich, Germany)). It should be noted that the initial bulk films were prepared from solutions of the three samples in toluene (5% *w*/*v*), where the solvent was left to slowly evaporate leading to bulk self-assembly of the terpolymers. More details are given in the main manuscript.

The synchrotron beam source from the beamline BL23A of the National Synchrotron Radiations Research Center (NSRRC) was used for the SAXS experiments at which a mirror and monochromatic to the energy of 10 keV by a germanium (111) double-crystal monochromator was used to vertically focus the incident X-ray beam. The wavelength of the X-ray beam was 1.24 Å. The beam stop was a round tantalum disk 4 mm in diameter. A MAR CCD X-ray detector (Rayonix L.L.C., Evanston, IL, USA) was used to collect the two-dimensional (2D) SAXS patterns. The films studied for the SAXS experiments were parts from those prepared for the TEM studies prior ultra-cryomicrotoming.

## 3. Results and Discussion

**Molecular and Thermal Characterization.** The SEC, VPO and ^1^H-NMR results, as evident in Table 2, justify that the three different terpolymers can be considered well-defined materials.

Differential scanning calorimetry (DSC) experiments were carried out in order to verify the existence of the characteristic glass transition temperatures (T_g_) for the three chemically different segments. The DSC thermographs are evident in Supplementary Materials Figures S7–S9, and T_g_ endothermal peaks were observed only for PS and PDMS segments, with values corresponding to low-molecular-weight PS (65 to 74 °C) and PDMS (−121 to −119 °C) segments as reported in the literature [78,79,80]. The absence of the PB glass transition temperature is attributed to the very low number average molecular weight (varying in the three samples, from 1.300 to 1.900 g/mol) and also suggests that actually a two-phase system might be observed during microphase separation studies. Supplementary Materials Table S2 in indicates the entanglement (M_e_) and critical (M_c_) average molecular weights for each block, according to the literature, in order to justify the absence of any glass transition, especially for such low-molecular-weight PB segments, which are well below the preferred M_e_ of 2.600 g/mol (References [1,2,3] in the Supplementary Materials). Despite the fact that the PS according to Supplementary Materials Figures S7–S9, for all three samples, is well above room temperature (ranging from 65 to 74 °C), the fact that its molecular weight is well above that of the PB does not lead to the probability of possible entrapment of the system in a non-preferred morphology.

**Structure/Properties Relationship.** The morphological characterization of the synthesized samples was achieved via transmission electron microscopy (TEM) and small angle X-ray scattering (SAXS). All samples were casted in toluene, which can be considered a good solvent for all blocks, showing preference to the PS, then to the PB and finally to the PDMS segments, according to the solubility parameters of the three blocks relative to that of toluene. As it is evident in the literature [40,41], the solubility parameter δ of the segments is δ_PS_ = 9.1 cal/cm^3^, δ_PB_ = 8.4 cal/cm^3^ and δ_PDMS_ = 7.3 cal/cm^3^, respectively, while, for toluene, the value is δ_tol_ = 8.9 cal/cm^3^. Based on the aforementioned values and the literature [51], it is evident that PS microphase separates faster from the other two blocks and the final self-assembly in the corresponding three-phase structures occurs in a second step.

After the complete solvent evaporation, the bulk samples were ultra-cryomicrotomed in order to retrieve ultra-thin sections (with thickness below 30 nm due to the low molecular characteristics in order to achieve at least one unit cell in dimension, but no more than two, if complex morphologies were adopted) suitable for the subsequent morphological characterization.

Since one block is a polydiene, staining process with vapors of osmium tetroxide (OsO_4_, Science Services, 2% *w*/*v* in water) was employed, in order to increase the electron density through crosslinking and thus enhance the image contrast of PB relative to the PS.

It should be mentioned that all samples exhibit extremely low average molecular weights, and, therefore, the microphase separation of these systems was conducted without performing thermal annealing for all cases. Additionally, for comparison reasons, the terpolymers were studied before and after staining with OsO_4_, in order to justify the existence of the third phase corresponding to PB in all cases.

According to Supplementary Materials Table S2, all segments in all three final terpolymers exhibit number average molecular weights well below the preferred entanglement (M_e_) average molecular weights (2.600 g/mol for PB, 16.500 g/mol for PS and 10.000 g/mol for the PDMS), leading to the conclusion that the chains are already stretched (without any entanglements), and therefore thermal annealing will not lead to any advancement of the already well-organized and adopted structures for all three terpolymers. Therefore, all the results from TEM and SAXS characterization correspond exclusively to unannealed samples. Furthermore, the values of the three different Flory–Huggins interaction parameters (*χ*_PS/PB_, *χ*_PB/PDMS_ and *χ*_PS/PDMS_) are also given in Supplementary Materials Table S3, where the details of their theoretical calculations are provided based on equations known from the literature (References [4,5,6,7] in the Supplementary Materials).

**Figure 1:**
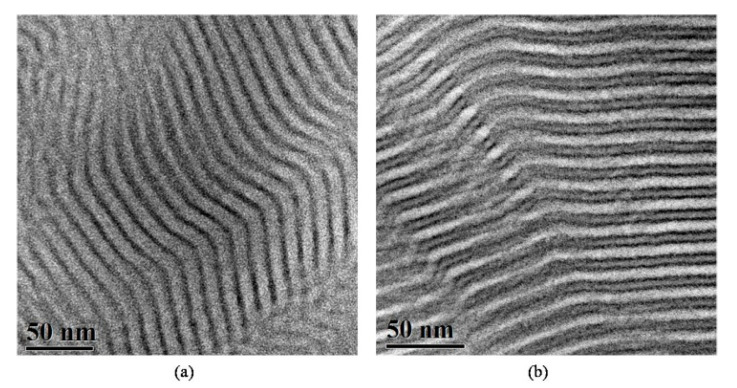
TEM images of the PS-*b*-PB_1,4_-*b*-PDMS terpolymer (sample 1). (**a**) Unstained sections where a two-phase alternating lamellae morphology is evident, with dark regions corresponding to PDMS and gray to the mixed PS/PB_1,4_ segments, respectively. (**b**) Sections stained for 60 min with vapors of OsO_4_, leading to a very distinct three-phase four-layer alternating lamellae structure. Black corresponds to PB, white to PS and gray to PDMS domains.

In Figure 1, TEM images from both unstained and stained sections of sample 1 (PS-*b*-PB_1,4_-*b*-PDMS sequence with total ${\bar{M}}_{n}^{\mathrm{tot}}$ of 11.000 g/mol) are shown. As expected, before staining, the microphase separation indicated a two-phase alternating lamellae morphology, due to the fact that PS and PB_1,4_ segments are miscible, since they possess similar electron density. As a result, PS and PB_1,4_ have a gray color, while the PDMS segments appear darker in the TEM micrograph, due to enhanced scattering cross-section of the silicon-containing block (Figure 1a). After staining with vapors of OsO_4_, the PB_1,4_ domains appear darker due to crosslinking and enhancement of the PB electron density, while the other blocks (i.e., PS and PDMS) appear lighter (white and gray, respectively), as evident in Figure 1b. From Figure 1b, for the PB_1,4_ segment, the layer thickness was calculated at approximately ~1.9 nm (×2), while, for the PDMS and the PS segments, the thickness was ~3.8 and ~6.1 nm, respectively, leading to a total approximate value of 14 nm for the d-spacing of the unit cell, as calculated by the TEM instrument capability (after correct calibration with specific standards at 200 kV, which is the accelerating voltage used for the TEM experiments).

According to the literature, in most cases, to obtain a three-phase four-layer lamellae morphology, a composition ratio of 1/1/1 between the three blocks and equivalent interaction parameters χ between all components (*χ*_AB_ ≈ *χ*_BC_ ≈ *χ*_AC_) are required [81]. There are a few discrepancies from that rule reported in the literature for samples of the PI-*b*-PS-*b*-PMMA sequence, as reported by Epps and his research group [58].

For sample 1, the block volume fraction ratio PS/PB/PDMS is approximately equal to 5/1/4, and the Flory–Huggins interaction parameter values correspond to the inequality: *χ*_PS/PB_ < *χ*_PB/PDMS_ < < *χ*_PS/PDMS_. Actually, microphase separation between the three chemically different blocks was not expected, as already mentioned in the DSC results.

**Figure 2:**
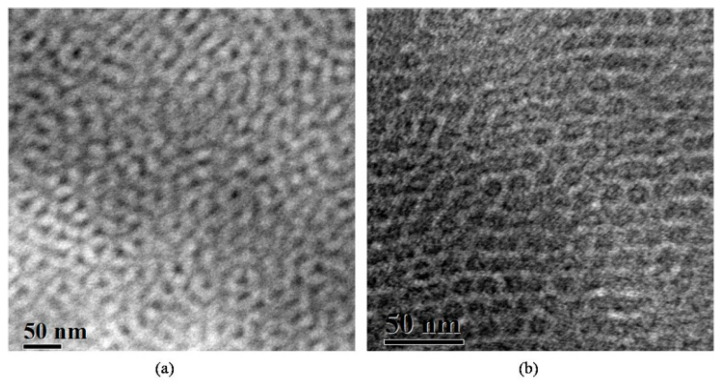
TEM images of the PS-*b*-PB_1,4_-*b*-PDMS terpolymer (sample 2). (**a**) Unstained sections where a 2-phase morphology consistent with the DG structure is evident. Dark areas correspond to PDMS segments and gray to the PS/PB_1,4_ mixed blocks. The existence of high symmetry projections (three-fold and four-fold) justifies the cubic morphology. (**b**) Sections stained for 60 min with vapors of OsO_4_, leading to a very distinct three-phase cubic microdomain structure consistent with the core–shell gyroid morphology. Black corresponds to PB segments, white to PS and gray to PDMS domains.

Sample 2 (of the PS-*b*-PB_1,4_-*b*-PDMS sequence), with a total ${\bar{M}}_{n}^{\mathrm{tot}}$ of 13.100 g/mol and block volume fraction ratio PS/PB/PDMS approximately equal to 5/1/4 (similar to that of sample 1), led to different morphological results when unstained and stained sections with vapors of OsO_4_ for 60 min (Figure 2a,b respectively) were studied. Again, after staining, a three-phase system occurs, but it is not a three-phase four-layer lamellae structure, as can be observed in Figure 2b.

The TEM image prior to staining indicates a structure very consistent to a cubic morphology relevant to the double gyroid morphology, since [111] and [100] high symmetry projections are evident (Figure 2a). A two-phase contrast is observed, where the darker phase corresponds to the PDMS (networks) and the gray domains (matrix) correspond to the mixture of PS and PB_1,4_. In this case, the mass fraction of PDMS is approximately 0.42 (leading to a volume fraction of 0.41), which is very close to that reported in the literature, by our group, where diblock copolymers of PS-*b*-PDMS sequences were studied and the DG morphology was observed for volume fractions of the PDMS segments equal to ~0.41 [82]. Moreover, the PDMS occupied the networks in the PS matrix in the copolymer, as is evident in the TEM image in Figure 2a. After staining with OsO_4_ for 60 min, three phases are observed (Figure 2b). High symmetry projections are evident corresponding to three-fold (hexagons) and four-fold (squares) symmetries, leading to the fact that a core–shell gyroid morphology is adopted where the networks are occupied by PS (white), covered peripherally by the lower in concentration PB (dark regions) in the matrix of PDMS (gray areas). Such images have already been reported in the literature for higher-molecular-weight samples and different sequences [65,83].

**Figure 3:**
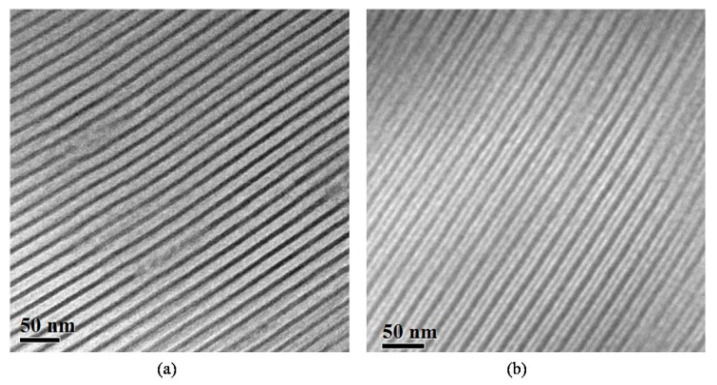
TEM images of the PB_1,4_-*b*-PS-*b*-PDMS terpolymer (sample 3). (**a**) Unstained sections where a two-phase alternating lamellae morphology is evident, with dark regions corresponding to PDMS and gray to the mixed PS/PB_1,4_ segments, respectively. (**b**) Sections stained for 30 min with vapors of OsO_4_, leading to a distinct three-phase four-layer alternating lamellae structure, as in sample 1, where the block sequence is different. Black corresponds to PB, white to PS and dark gray to PDMS domains.

For sample 3 (of the PB_1,4_-*b*-PS-*b*-PDMS sequence), with a total ${\bar{M}}_{n}^{\mathrm{tot}}$ of 14.000 g/mol and block volume fraction ratio PS/PB/PDMS approximately equal to 4/1/5, TEM images from the unstained and stained sections with OsO_4_ are given in Figure 3.

Two-phase lamellae are observed for the unstained sections, leading again to white layers of the mixed phase of PS and PB and darker PDMS layers (Figure 3a). After staining with OsO_4_, a three-phase four-layer lamellae structure is evident (Figure 3b), with a color-contrast sequence different to that observed for sample 1 (Figure 1b), since, in this case, the middle block is the PS and not the PB_1,4_. This contrast difference is attributed to the decreased staining time with OsO_4_ vapors (30 min instead of 60 min, as in the case of samples 1 and 2). This observation is very important, since it leads to the conclusion that the crosslinking of PB_1,4_ segments with OsO_4_ strongly depends on the staining duration time.

Despite the fact that the contrast between the PB and PDMS layers for sample 3 after staining is minimal (almost dark contrast for both blocks), the difference in layer thickness also clearly justifies the importance of thorough and analytical molecular characterization, since, for the PB_1,4_ segment, the layer thickness was calculated at approximately ~2.4 nm, while, for the PS and the PDMS segments, the thickness was ~2.8 nm (×2) and ~4.8 nm, respectively, leading to a total approximate value of ~13 nm for the d-spacing of the unit cell, as calculated by the TEM instrument capability. Again, in this sample, as in sample 1, a three-phase four-layer lamellae morphology is evident without the composition ratio between the three blocks being equal to 1/1/1 and without having equivalent interaction parameters *χ* between all components (*χ*_AB_ ≈ *χ*_BC_ ≈ *χ*_AC_) [81].

**Figure 4:**
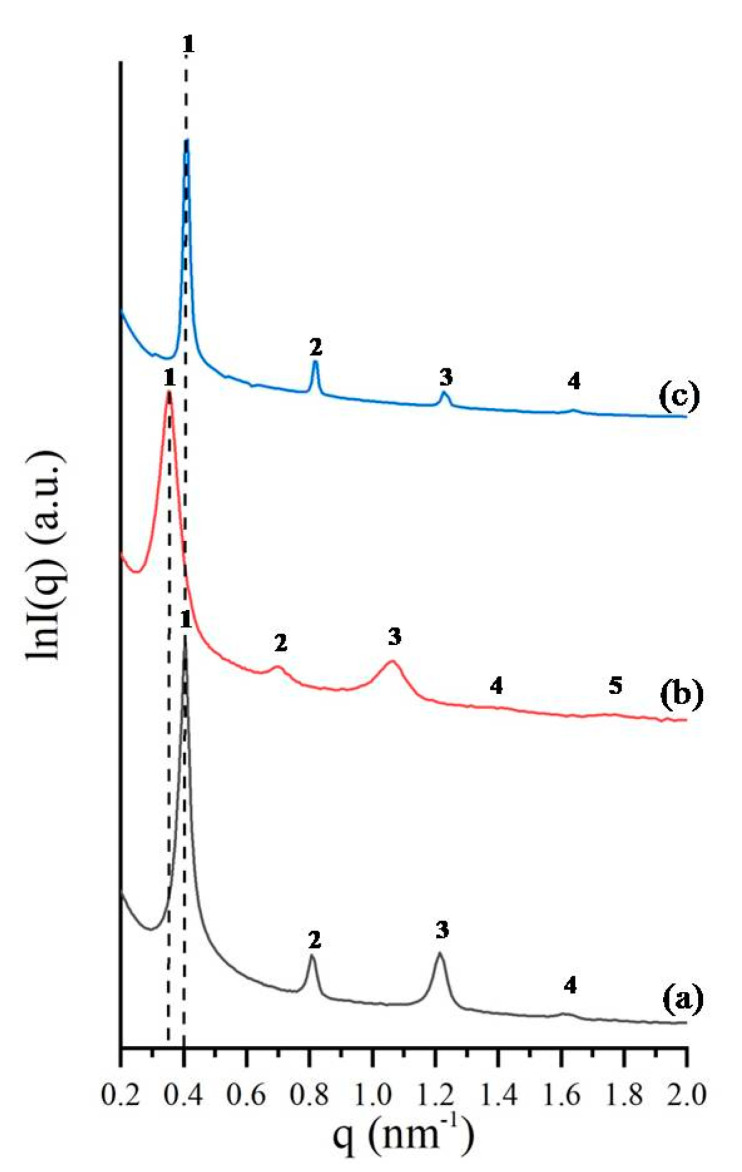
SAXS plots of lnI(q) versus q for the three different triblock terpolymer samples: (**a**) PS-*b*-PB_1,4_-*b*-PDMS (sample 1), (**b**) PS-*b*-PB_1,4_-*b*-PDMS (sample 2) and (**c**) PB_1,4_-*b*-PS-*b*-PDMS (sample 3).

In order to further investigate the obtained morphologies, small-angle X-ray scattering (SAXS) experiments were made and are illustrated in Figure 4. It is clear from the lnI(q) vs. q-plots that four and five peaks are visible, indicating that these samples, despite the considerably low number average molecular weight values, after casting even without any type of annealing, are well ordered in bulk, as evident also from the TEM images in Figure 1, Figure 2 and Figure 3, and as mentioned already, due to their molecular characteristics below the M_e_ for all segments. According to the International Tables for X-Ray Crystallography [84], the lamellae structure has a pm space group with permitted reflections [100], [200], [300], [400].

In line with the aforementioned requirement, the characteristic one-dimensional SAXS profile for sample 1 (Figure 4a) further justified the formation of a lamellar phase, since the relative q-values of the observed peaks correspond to a ratio of 1:2:3:4. Based on the value of the primary permitted peak and through the equation q = 2π/d, the domain spacing was calculated equal to d = 15 nm, which is in a good agreement with TEM results (~14 nm).

For sample 2, the SAXS plot (Figure 4b) justified the formation of lamellae morphology and not a cubic structure as evidenced by the TEM images (Figure 2). The relative q-values of the observed peaks correspond again to a ratio of 1:2:3:4:5, corresponding to a very well-ordered lamellar structure. Such an observation has been made by our group [65] in a previous publication, where, for a triblock terpolymer of the PS-*b*-PB_1,4_-*b*-PI_3,4_ sequence (where PI_3,4_ is poly(isoprene) with high 3,4-microstructure, ~60%), the TEM images indicated the co-existence of three-phase four-layer lamellae, together with core–shell-DG, but the SAXS plot exhibited peaks corresponding exclusively to pm symmetry. In order to verify the SAXS results for sample 2, additional sections were taken and observed with TEM, in order to justify the existence of alternating lamellae together with the observed core–shell gyroid structure (Figure 2). Furthermore, it might be suggested that the system is close to a phase boundary; therefore, temperature change might lead to structural differentiations. We believe that the results obtained are very novel, and therefore additional samples will be synthesized in order to verify and justify the lowest possible dimensions that can be reached for such triblock terpolymer systems. Based on the value of the primary permitted peak and through the equation q = 2π/d, the domain spacing of the unit cell was calculated equal to d = 16 nm.

For sample 3, where the block sequence is different compared to samples 1 and 2, the lamellae morphology evident from TEM (Figure 3) is confirmed from the SAXS plot (Figure 4c). In this case, as in sample 1, the relative q-values of the observed peaks correspond to a ratio of 1:2:3:4. Based on the value of the primary permitted peak and through the equation q = 2π/d, the domain spacing was calculated equal to d = 16 nm, which is in a good agreement with TEM results (~13 nm).

## 4. Conclusions

To conclude, three novel and narrow dispersed triblock terpolymers with very low total number average molecular weights were synthesized via anionic polymerization, and their self-assembly was studied with TEM and SAXS techniques. The block sequence was either PS-*b*-PB_1,4_-*b*-PDMS (2 samples) or PB_1,4_-*b*-PS-*b*-PDMS (1 sample). Such terpolymers have never been reported in the literature, and this is the first time that terpolymers exhibiting very low molecular characteristics have self-assembled in bulk, exhibiting well-defined morphologies (three-phase four-layer lamellae and core–shell gyroid) without any thermal annealing processes. The TEM images exhibited two phases prior to the staining of the PB segments with vapors of OsO_4_ due to mixing of the PS and PB domains, whereas, after staining for 60 min, the three phases were well illustrated. The TEM results are supported by the SAXS plots, since at least four prominent peaks are evident in all three samples. This study is very well supported by the already published article by Sinturel, Bates and Hillmyer [85], where the importance of low-molecular-weight diblock copolymers with high χ values in microelectronics industry and nanotechnology manufacturing methods with dimensions of sub-10 nm is elaborated. Since we have shown that sub-16 nm dimensions can be reached in our samples, we strongly believe that, under careful synthetic routes, even lower dimensions will be reached, thus enhancing the importance of these triblock terpolymers in nanopatterning applications.

## Figures and Tables

**Table 1:** Linear triblock terpolymer block sequences studied in the literature [23,24,25,26,27,28,29,30,31,32,33,34,35,36,37,38,39,40,41,42,43,44,45,46,47,48,49,50,51,52,53,54,55,56,57,58,59,60,61,62,63,64,65,66,67,68,69,70,71,72,73,74]. The total number average molecular weight range is given, together with the annealing conditions (if any) and the observed morphologies, in all cases.

No.	Block Sequence	Molecular Weight Range (kg/mol)	Annealing	Observed Morphology	References
1	PS-*b*-PB_1,4_-*b*-P4VP	74–113	60 °C, 14 h	Ball in Box, LAM	[23,24]
2	PS-*b*-PA-*b*-PI	131–198	n/a	LAM, C_h_C	[25]
3	PS-*b*-PB-*b*-P4VP	65–210	n/a	Ball in Box, LAM	[26]
4	PI-*b*-PS-*b*-P2VP	36–279	120 °C, 10 days	LAM, OTDD, CYL, SPH	[20,21,22,27]
5	PS-*b*-PI-*b*-P2VP	43	120 °C, 7 days	LAM, C_h_C	[19]
6	PS-*b*-P2VP-*b*-PB	111–258	n/a	n/a	[28]
7	PS-*b*-PI-*b*-P2VP	196–201	120 °C, 7 days	OTDD, LAM	[29]
8	PS-*b*-PB_1,2_-*b*-PMMA and PS-*b*-PEB-*b*-PMMA	225–245 and 226–248	85 °C, 2 days 150 °C, 2 h/4 h/6 h and 160 °C, 5 days	LAM, CR, LC, LS	[30,31]
9	PS-*b*-PB_1,2_-*b*-PMMA	206–218	100 °C, 2 days 170 °C, 2 h/4 h/6	C_h_C, C_a_C, HEL	[32]
10	PS-*b*-PB_1,2_-*b*-PCL	105–137	n/a	n/a	[33]
11	PS-*b*-PB_1,2_-*b*-PMMA and PS-*b*-PEB-*b*-PMMA	117–245	160 °C, 4 h	KP, LAM, LC	[34]
12	PB_1,2_-*b*-PS-*b*-PMMA	192	100 °C, 2 days 170 °C, 6 h	CYL_T_	[35]
13	PS-*b*-PB_1,2_-*b*-PMMA and PS-*b*-PEB-*b*-PMMA	78–140 and 80–124	185 °C, 6 h and 185 °C, 2 h/4 h/6 h	C_h_C, HEL, C_a_C, uC_i_C, S_o_C	[36]
14	PS-*b*-PVP-*b*-PtBMA	293	n/a	LAM	[37]
15	PS-*b*-PB_1,2_-*b*-PMMA and PS-*b*-PEB-*b*-PMMA	88–241 and 90–242	170 °C, 10 days	S_o_S	[38]
16	PI_1,4_-*b*-PB_1,2_-*b*-PS	48–74	60 °C, 2 days 120 °C, 3 h	two-phase morphology	[39]
17	PS-*b*-PB_1,2_-*b*-PMMA	215	120 °C, 3–5 days	LAM, HPC	[40]
18	PS-*b*-PB_1,2_-*b*-PMMA and PS-*b*-PEB-*b*-PMMA	121 and 122	185 °C, 2–6 h	LAM, KP	[41]
19	PH-*b*-P2T-*b*-PF	61	n/a	LAM	[42]
20	PI_1,4_-*b*-PS-*b*-PDMS	41	n/a	CSG	[43]
21	PS-*b*-PB_1,2_-*b*-P2VP and PB_1,2_-*b*-PS-*b*-P2VP	62–137 and 84–210	150 °C, 6 h	LAM, C_h_C, CSG	[44]
22	PS-*b*-PI-*b*-PDMS	59	n/a	n/a	[45]
23	PS-*b*-PB_1,2_-*b*-P2VP	71	150 °C, 6 h	CSG	[46]
24	PS-*b*-PI-*b*-PEO	19–30	80–225 °C, 0.5 h–120 h	LAM, C_h_C, CSG, PLS, SPL	[47]
25	PS-*b*-PEB-*b*-PMMA	73–123	170 °C, 6 h	LAM, LC, KP	[48]
26	PI-*b*-PS-*b*-PEO	13–22	n/a	LAM, Fddd (O^70^)	[49]
27	PI-*b*-PS-*b*-PVME	32	n/a	LAM	[50]
28	PB_1,4_-*b*-PS-*b*-PI_3,4_ and PS-*b*-PB_1,4_-*b*-PI_3,4_	85–149 and 133	130 °C, 7 days	LAM, HPC	[51]
29	PS-*b*-P2VP-*b*-PtBMA	76–140	n/a	C_h_C, CSG, LAM, PL, UL	[52]
30	PI-*b*-PS-*b*-PEO	13–25	n/a	Q^230^, LAM, O^70^, Q^214^	[53]
31	PI-*b*-PS-*b*-PEO	19–43	n/a	LAM, O^70^	[54]
32	PS-*b*-PFS^a^-*b*-PMMA	101–110	120 °C, 2 h 180 °C, 36 h	HEL/S_o_C, S_o_S	[55]
33	PS-*b*-PB-*b*-PMMA	170	100 °C, 1 day 170 °C, 1 day	dHEL	[56]
34	PI-*b*-PS-*b*-PFS^b^	82	150 °C, 4 days	CYL_T_	[57]
35	PS-*b*-PI-*b*-PMMA	13.5–31	n/a	LAM, Q^214^	[58]
36	PB-*b*-P2VP-*b*-PtBMA	61–165	50 °C, 24 h 130 °C	LAM/CSG, C_h_C, LAM, S_o_C, HEL_o_C	[59]
37	PI-*b*-PS-*b*-P2VP	26–150	150 °C, 7 days	LAM, UL	[60]
38	PI-*b*-PS-*b*-P4VP	77	n/a	HPC	[61]
39	PB-*b*-P2VP-*b*-PtBMA	110	n/a	TPL	[62]
40	PS-*b*-PB_1,2_-*b*-PCHD_1,4_ and PB_1,2_-*b*-PS-*b*-PCHD_1,4_	29–32 and 39	110 °C, 7 days	LAM, C_h_C	[63]
41	PS-*b*-PB-*b*-PtBMA	57–148	n/a	C_h_C, CSG, LAM, LC	[64]
42	PS-*b*-PB_1,4_-*b*-PI_3,4_	80–103	130 °C, 7 days 150 °C, 5 days	LAM, LAM/CSG	[65]
43	PI-*b*-PS-*b*-P2VP	122–124	150 °C, 5 days	GS, LC	[66]
44	PI-*b*-PS-*b*-P2VP	223–264	150 °C, 5 days	LS, HPC	[67]
45	PS-*b*-PB_1,4_-*b*-PI_3,4_	35–43	120 °C, 5 days	LAM	[68]
46	PI-*b*-PS-*b*-P2VP	136–146	150 °C, 5 days	LAM	[69]
47	PI-*b*-PS-*b*-P2VP	84	240 °C, 3 h	SPH/CYL	[70]
48	PS-*b*-P2VP-*b*-PEO	32–161	130 °C, 5 days	LAM, HPC	[71]
49	PS-*b*-PI-*b*-PMMA and PI-*b*-PS-*b*-PMMA	171 and 95–318	80 °C, 2 days 150 °C, 6 h	HEL_o_C, CSG	[72]
50	PS-*b*-PI-*b*-PMMA	171–324	80 °C, 2 days 150 °C, 6 h	‘planetlike’, HEL_o_C, S_o_C	[73]
51	PS-*b*-PB-*b*-PMMA	74–202	n/a	LAM, LS, LC	[74]

PS, polystyrene; PB, poly(butadiene); PA, poly[(4-vinylbenzyl)dimethylamine]; P4VP, poly(4-vinylpyridine); P2VP, poly(2-vinylpyridine); PI poly(isoprene); PDMS, poly(dimethylsiloxane); PMMA, poly(methyl methacrylate); PtBMA, poly(tert-butyl methacrylate); PEO, poly(ethylene oxide); PEB, poly(ethylene-co-butylene); PCHD, poly(cyclohexadiene); PFS^a^, poly(dimethylsilaferrocenophane); PFS^b^, poly(ferrocenylsilane); PCL, poly(ε-caprolactone); PH, poly(2-hydroxyethyl methacrylate); PF, poly[2 (perfluorobu-tyl) ethyl methacrylate]; P2T, poly(tert-butyl methacrylate); PVME, poly(vinyl methyl ether); n/a: not available. LAM, 3-phase 4-layer lamellae; CYL, cylindrical structure; OTDD, ordered tricontinuous double diamond; SPH, spheres; C_h_C, hexagonally arranged core shell cylinders; LC, cylinders on lamellae; CR, rings on cylinders; LS, spheres on lamellae; HEL, helical morphologies; KP, knitting pattern; CYL_T_, tetragonally packed cylinders; S_o_C, spheres on cylinders; C_a_C, cylinder at cylinder; uC_i_C, perforated cylinder in cylinder; S_o_S, spheres on spheres; HPC, hexagonally packed cylinders; CSG, core shell gyroid; Fddd (O^70^), non-cubic orthorhombic network morphology; Q^230^, core shell double gyroid; Q^214^, alternating gyroid; LAM/CSG, coexistence of lamellae and gyroid; PLS, pillared lamellar structure; SPL, semi-perforated lamellae; PL, perforated lamellae; UL, undulated lamellae; dHEL, double helical structure; GS, sphere in gyroid structure; HEL/S_o_C, coexistence of helical and spheres on cylinders; HEL_o_C, helices on cylinders; TPL, tetragonally perforated lamellae; SPH/CYL, coexistence of spheres and cylinders.

**Table 2:** Molecular characteristics of all blocks of the three triblock terpolymers synthesized.

Sample	A-*b*-B-*b*-C	${{\bar{M}}_{n}^{A}\;}^{\left(a\right)}$ (g/mol)	${{\bar{M}}_{n}^{B}\;}^{\left(a\right)}$ (g/mol)	${{\bar{M}}_{n}^{C}\;}^{\left(a\right)}$ (g/mol)	${{\bar{M}}_{n}^{tot}\;}^{\left(a\right)}$ (g/mol)	Đ^SEC (b)^	*f*_A_^(c)^	*f*_B_^(c)^	*f*_C_^(c)^	PB_1,4_^(c)^ (%)	PB_1,2_^(c)^ (%)
1	PS-*b*-PB_1,4_-*b*-PDMS	5.200	1.300	4.500	11.000	1.04	0.52	0.09	0.39	90	10
2		6.300	1.700	5.100	13.100	1.04	0.48	0.10	0.42	89	11
3	PB_1,4_-*b*-PS-*b*-PDMS	1.900	6.100	6.000	14.000	1.04	0.11	0.42	0.47	93	7

^(a)^ VPO in toluene at 45 °C. ^(b)^ Dispersity (Đ) calculated from SEC in ΤHF at 30 °C. ^(c)^ Mass fractions for the three blocks and 1,4-/1,2-microstructure percentages for the PB segments calculated from ^1^H-NMR in CDCl_3_ at 25 °C.

## Supplementary Materials

The following are available online. Scheme S1: Synthetic routes of (a) PS-*b*-PB_1,4_-*b*-PDMS and (b) PB_1,4_-*b*-PS-*b*-PDMS triblock terpolymers. Figure S1: SEC chromatographs of the PS precursor (red), the diblock intermediate product of PS-*b*-PB_1,4_ type (blue) and the final triblock terpolymer (sample 1) of the PS-*b*-PB_1,4_-*b*-PDMS type (black). Figure S2: SEC chromatographs of the PS precursor (red), the diblock intermediate product of PS-*b*-PB_1,4_ type (blue) and the final triblock terpolymer (sample 2) of the PS-*b*-PB_1,4_-*b*-PDMS type (black). Figure S3: SEC chromatographs of the PS precursor (red), the diblock intermediate product of PS-*b*-PB_1,4_ type (blue), the unfractionated triblock terpolymer (green) and the final triblock terpolymer (sample 3) of the PB_1,4_-*b*-PS-*b*-PDMS type (black). Figure S4: ^l^H-NMR spectrum of the PS-*b*-PB_1,4_-*b*-PDMS triblock terpolymer (sample 1). Figure S5: ^l^H-NMR spectrum of the PS-*b*-PB_1,4_-*b*-PDMS triblock terpolymer (sample 2). Figure S6: ^l^H-NMR spectrum of the PB_1,4_-*b*-PS-*b*-PDMS triblock terpolymer (sample 3). Table S1: The type and number of protons per monomeric unit of each block, as well as the chemical shifts, are presented in order to comprehend the ^1^H-NMR spectra of the terpolymers. Table S2: Entanglement (M_e_) and critical (M_c_) average molecular weights for the three different blocks in the synthesized final triblock terpolymers. Figure S7: DSC thermograph of the PS-*b*-PB_1,4_-*b*-PDMS triblock terpolymer (sample 1), where only the two T_g_s of PS and PDMS are evident. Figure S8: DSC thermograph of the PS-*b*-PB_1,4_-*b*-PDMS triblock terpolymer (sample 2), where only the two T_g_s of PS and PDMS are evident, together with the T_c_ and T_m_ of PDMS. Figure S9: DSC thermograph of the PB_1,4_-*b*-PS-*b*-PDMS triblock terpolymer (sample 3), where only the two T_g_s of PS and PDMS are evident together with the T_c_ and T_m_ of PDMS. Table S3: Flory–Huggins *χ* interaction parameter for PS/PDMS and PS/PB_1,4_.
