# Supplementary material for: Self-Assembly of Low-Molecular-Weight Asymmetric Linear Triblock Terpolymers: How Low Can We Go?
Source: Molecules. 2020 Nov 25;25(23):5527. doi: 10.3390/molecules25235527 (PMC7728154; doi:10.3390/molecules25235527)
Supplement: Supplementary file 1 [file molecules-25-05527-s001.pdf]

Self-Assembly of Low Molecular Weight Asymmetric Linear Triblock Terpolymers. How Low Can We Go?

Christina Miskaki[1], Ioannis Moutsios[1], Gkreti-Maria Manesi[1], Konstantinos Artopoiadis[1], Cheng-Yen Chang[2], Egor A. Bersenev[3,4], Dimitrios Moschovas[1,3], Dimitri A. Ivanov[3,4,5], Rong-Ming Ho[2] and Apostolos Avgeropoulos[1,3,*]

[1]Department of Materials Science Engineering, University of Ioannina, University Campus-Dourouti, 45110 Ioannina, Greece

[2]Department of Chemical Engineering, National TsingHua University, Hsinchu 30013, Taiwan

[3]Faculty of Chemistry, Lomonosov Moscow State University (MSU), GSP-1, 1-3 Leninskiye Gory, 119991 Moscow, Russia

[4]Institute of Problems of Chemical Physics, Russian Academy of Sciences, Chernogolovka, 142432 Moscow, Russia;

[5]Institut de Sciences des Matériaux de Mulhouse – IS2M, CNRS UMR7361, 15 Jean Starcky, Mulhouse 68057, France.

Supporting Information

The following data are given in the Supporting Information:

A). Synthesis of the Triblock Terpolymers

B). SEC and ^{1}H-NMR Molecular Characterization Results

C). DSC Thermal Characterization Results

D). Estimation of the Flory–Huggins Interaction Parameter (χ)

A). Synthesis of the Triblock Terpolymers

Anionic polymerization under high vacuum techniques, through sequential addition of monomers, was employed in order to prepare two samples of the PS-*b*-$PB_{1,4}$-*b*-PDMS type and one sample of the $PB_{1,4}$-*b*-PS-*b*-PDMS type.

Synthesis of PS-*b*-$PB_{1,4}$-*b*-PDMS

The reaction of styrene (purity 99%) with *sec*-BuLi (Sigma-Aldrich, 1.4 M in cyclohexane) was accomplished after 18h in room temperature using a non-polar solvent (benzene, purity 99,7%). Subsequently, the 1,3-butadiene (purity 99+%) was added to the solution and the reaction was kept at room temperature for 24h until all quantity of the second monomer was consumed. Following the specific synthetic procedure leads to high -1,4 microstructure (~92%) while a small percentage (~8%) is attributed to the vinyl content (-1,2 microstructure). In the third step thoroughly purified hexamethylcyclotrisiloxane (D_3) (purity 98%) was introduced to the solution and after 18h the ring opening of the D_3 took place. An equal quantity of a polar solvent (tetrahydrofuran, purity 99.9%) relative to that of the benzene, was added to the solution and after 4h at room temperature the solution was placed in -20°C for seven days under continuous stirring in order to successfully polymerize the D_3 with 100% yield as required in anionic polymerization. After completion of the polymerization the final triblock terpolymer was precipitated in a non-solvent (methanol, purity 99%) for all blocks and was vacuum-dried. Aliquots from the solution were taken during the three different polymerization steps, for molecular characterization purposes, in order to verify the successful synthesis of the homopolymer, the diblock and the final terpolymer. The reaction procedure for the PS-*b*-$PB_{1,4}$-*b*-PDMS type linear triblock terpolymers is illustrated in Scheme S1a (all reagents were purchased from Sigma-Aldrich Co., St Louis, MO, USA).

Synthesis of $PB_{1,4}$-*b*-PS-*b*-PDMS

In order to synthesize the triblock terpolymer of $PB_{1,4}$-*b*-PS-*b*-PDMS sequence a different synthetic route was adopted from that described above. The reaction of 1,3-butadiene with *sec*-BuLi was accomplished after 24h in room temperature using benzene as a solvent. A small quantity of THF was then added to the solution, in order to alter the aggregation degree, prior to the addition of the styrene and also to increase the initiation vs. propagation rate of the styrenic monomers from the $PB^{(-)}Li^{(+)}$macroinitiator. Subsequently, the styrene was introduced to the solution and after 18h the complete conversion of the second monomer was achieved. Finally, the third monomer (D_3) was also introduced to the solution following the same reaction route as already discussed previously. The synthetic route employed is given in Scheme S1b.

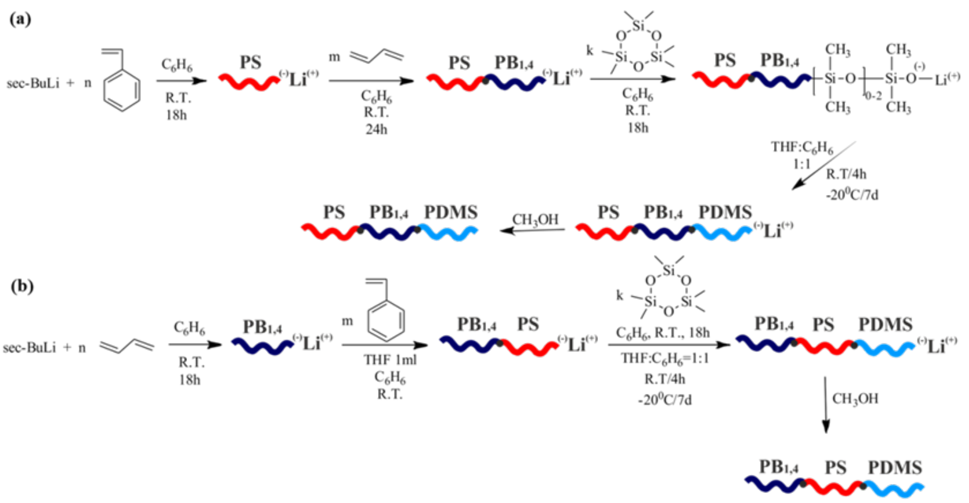

Scheme S1. Synthetic routes of a) PS-*b*-$PB_{1,4}$-*b*-PDMS and b) $PB_{1,4}$-*b*-PS-*b*-PDMS triblock terpolymers.

B). SEC and ^{1}H-NMR Molecular Characterization Results

In the following SEC chromatographs the initial block, the intermediate precursors and the final products are presented (Figures S1-S3). It is straightforward that the final terpolymers exhibit increased molecular and compositional homogeneity since the dispersity index (Đ) is well below 1.1 for all three cases.

For the third sample, in order to receive a purified final product, fractionation of the unpurified triblock was employed. Firstly, the unfractionated triblock terpolymer is dissolved to a good solvent for all blocks (toluene), creating a solution usually 1% w/v and the non-solvent (methanol) is added carefully to the diluted polymer mixture. Afterwards, the mixture is warmed up and then is added to a separation flask, until higher molecular weight macromolecules precipitate first, creating a lower layer and the by-products form an overlaying phase.

Finally, it is evident in the homopolymer and the diblock intermediates (for all three samples) that a small peak appears on the left of the main SEC peak corresponding to dimer product values resulting from the small quantity of oxygen incorporated in the aliquots during testing of the synthetic procedure process. This small peak is not evident in the final terpolymers since the termination reaction with methanol is conducted exclusively under high vacuum environment.

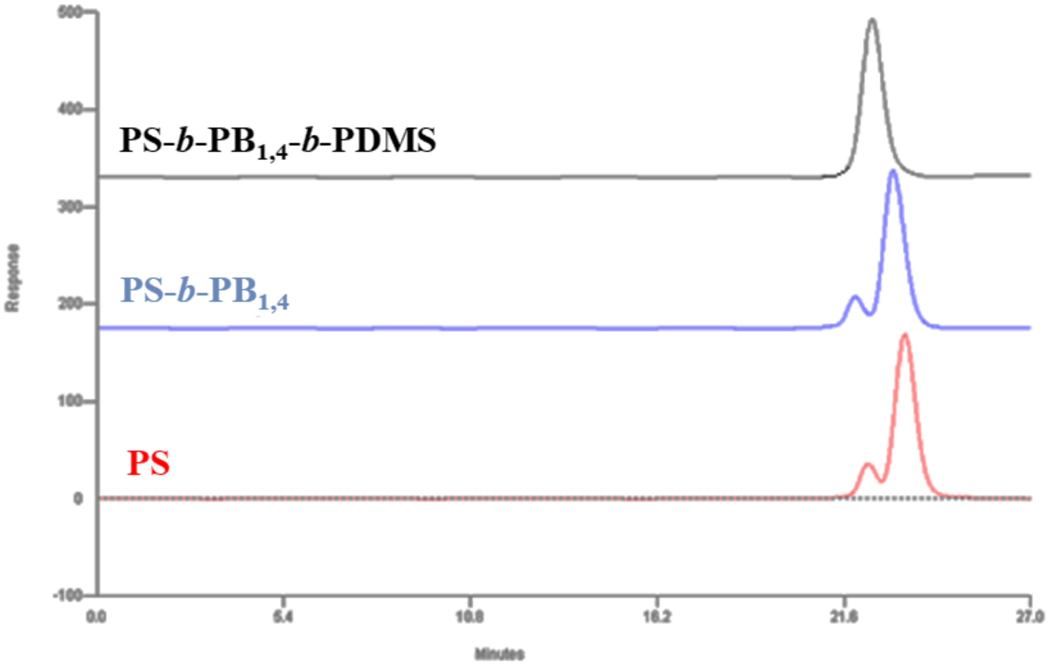

Figure S1. SEC chromatographs of the PS precursor (red), the diblock intermediate product of PS-*b*-$PB_{1,4}$ type (blue) and the final triblock terpolymer (sample 1) of the PS-*b*-$PB_{1,4}$-*b*-PDMS type (black).

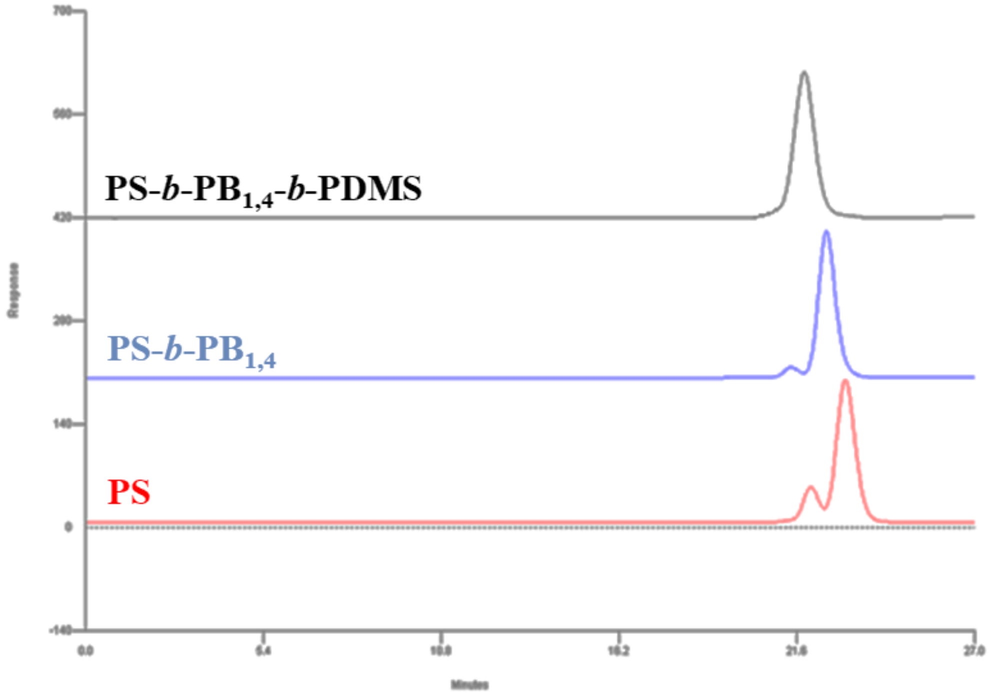

Figure S2. SEC chromatographs of the PS precursor (red), the diblock intermediate product of PS-*b*-$PB_{1,4}$ type (blue) and the final triblock terpolymer (sample 2) of the PS-*b*-$PB_{1,4}$-*b*-PDMS type (black).

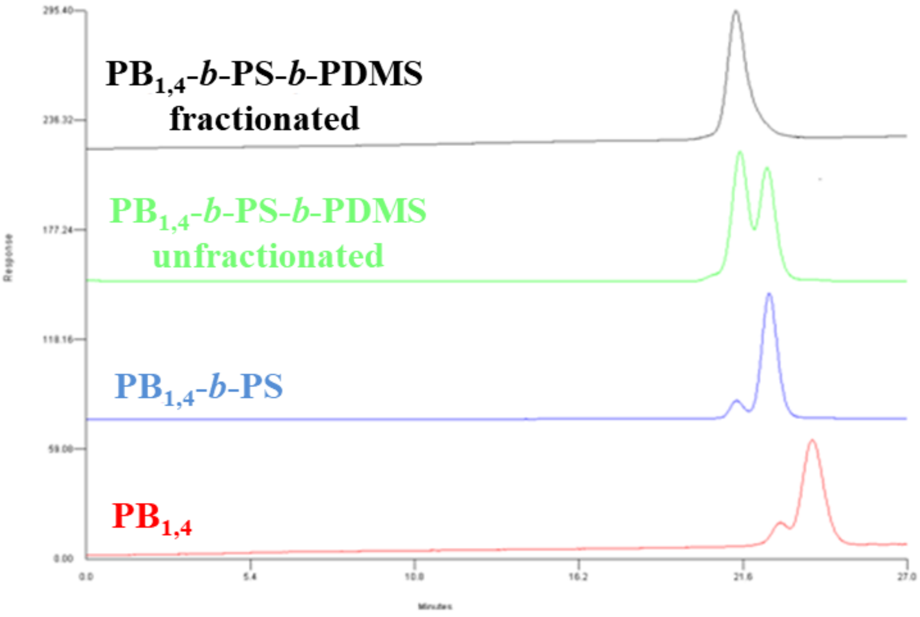

Figure S3. SEC chromatographs of the PS precursor (red), the diblock intermediate product of PS-*b*-$PB_{1,4}$ type (blue), the unfractionated triblock terpolymer (green) and the final triblock terpolymer (sample 3) of the $PB_{1,4}$-*b*-PS-*b*-PDMS type (black).

Through proton nuclear magnetic resonance spectroscopy (^{1}H-NMR), as shown in Figure S4-S6, the successful synthesis was verified, given that chemical shifts corresponding to specific protons of the monomeric units of PS, $PB_{1,4}$ and PDMS blocks are observed. The chemical shifts in the region 0-0.2 ppm are assigned to the six (6) protons of the two methyl groups in the monomeric units of PDMS, the chemical shifts in the region 5.3-5.5 ppm are attributed to the two (2) protons of the ethyl group in the monomeric units of $PB_{1,4}$ microstructure, in 5.0 and 5.6 ppm to the three (3) protons of the methyl groups in the monomeric units of $PB_{1,2}$ microstructure and in the region 6.7-7.7 ppm the chemical shifts correspond to the five (5) protons of the aromatic ring in the monomeric units of PS.

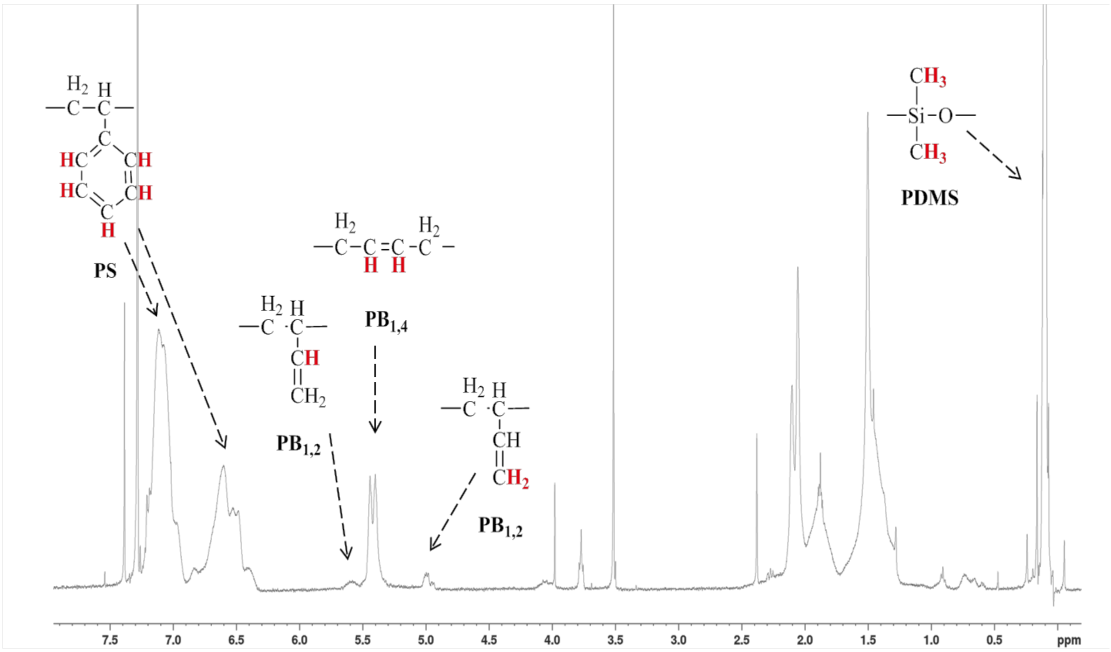

Figure S4. ¹H-NMR spectrum of the PS-*b*-$PB_{1,4}$-*b*-PDMS triblock terpolymer (sample 1).

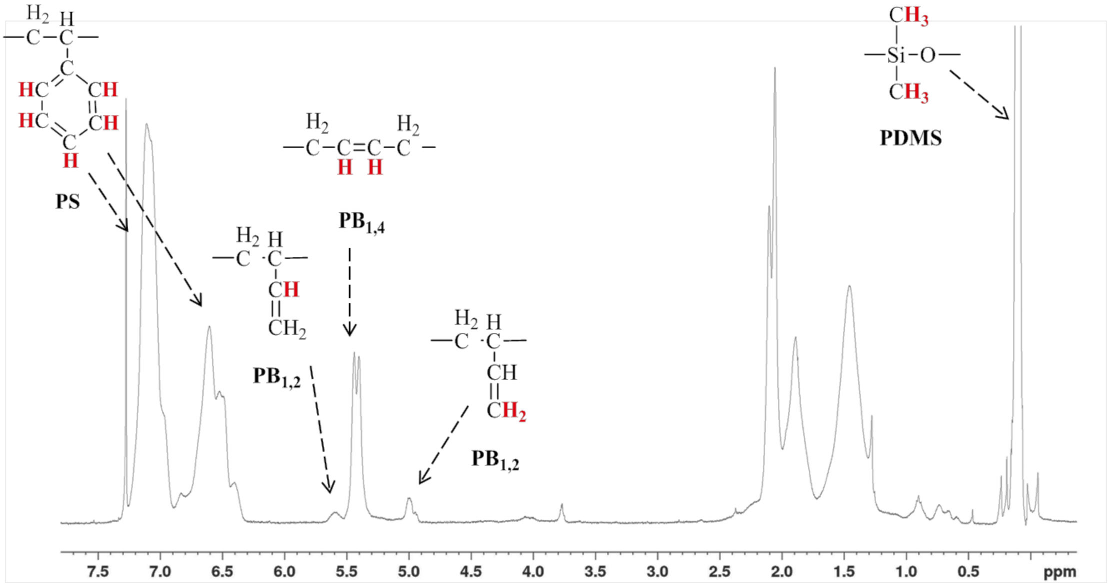

Figure S5. ¹H-NMR spectrum of the PS-*b*-$PB_{1,4}$-*b*-PDMS triblock terpolymer (sample 2).

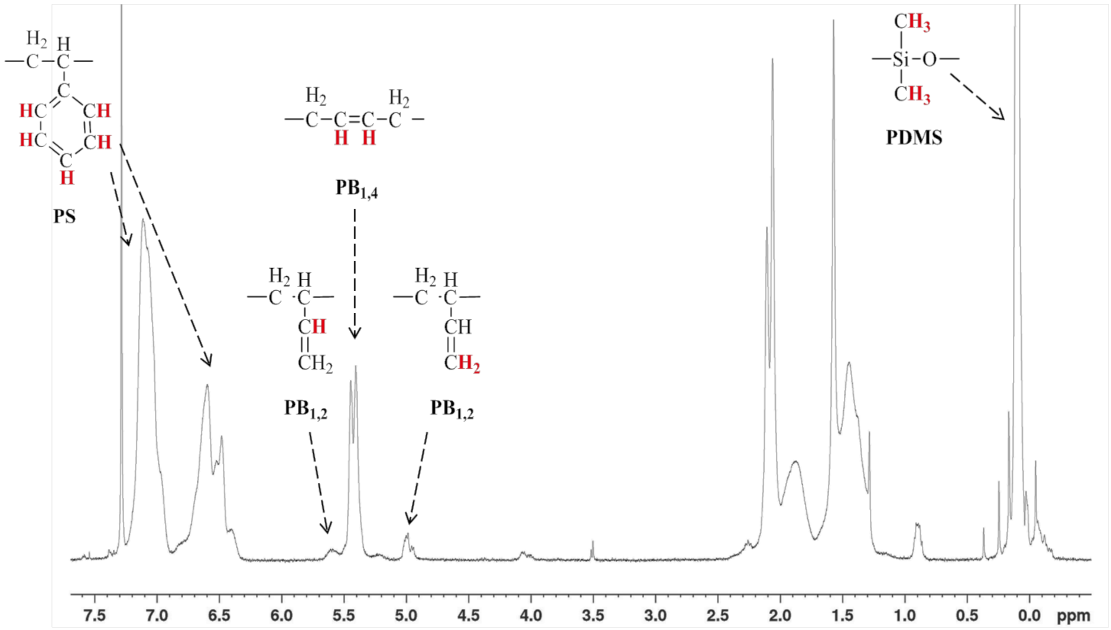

Figure S6. ^{1}H-NMR spectrum of the $PB_{1,4}$-*b*-PS-*b*-PDMS triblock terpolymer (sample 3).

Table S1. The type and number of protons per monomeric unit of each block as well as the chemical shifts, are presented in order to comprehend the ^{1}H-NMR spectra of the terpolymers.

Block	Geometric Isomerism	Type and Number of Protons	Chemical Shift (ppm)
PS		Aromatic (5)	6.30-7.37
PDMS		Aliphatic (6)	0.21
PB	1,4	Olefinic (2)	5.31
	1,2	Olefinic (1)<br>Olefinic (2)	5.60<br>4.95

C). DSC Thermal Characterization Results

In Figures S7-S9 the characteristic glass transition temperatures T_gs for the PS and PDMS blocks are observed. For sample 1 (Figure S7), two distinct T_gs are evident at -119°C and 66°C, corresponding to PDMS and PS segments of the final triblock terpolymer respectively. The lack of a distinct melting point of the PDMS is attributed to the low average molecular weight, which is significantly smaller from the entanglement molecular weight (M_e) as evident in Table S2.

For sample 2 (Figure S8) a similar behavior, regarding the T_gs of the PS (74°C) and PDMS (-120°C) segments, is observed. In addition, for the PDMS block, a crystallization point (T_c=-71°C) and a melting point (T_m=-47°C) are evident that leads to the conclusion that the PDMS segment is not completely amorphous. This fact can be attributed to the slightly increased average molecular weight of the PDMS block in comparison to sample 1, despite the fact that the average molecular weight of PDMS also in this sample is lower than the M_e (Table S2).

Similarly, for sample 3 (Figure S9), two distinct T_gs at -121°C and 65°C for the PDMS and PS blocks respectively, are observed. The crystallization (T_c=-88°C) and two melting points (T_m=-46 ^{o}C,-39°C) of the PDMS block are evident. In all cases the glass transition temperature for the $PB_{1,4}$ segments is absent, probably attributed to the very low average molecular weight, which is smaller than the corresponding M_e (Table S2). In Table S2 the relative M_e values for the three blocks consisting the triblock terpolymers of this study are given together with the approximate values of critical average molecular weight (M_c).

Table S2. Entanglement (M_e) and critical (M_c) average molecular weights for the three different blocks in the synthesized final triblock terpolymers [1-3].

**Block**	**M_e (g/mol)**	**M_e (g/mol) (preferred)**	**$M_c \approx 2M_e$ (g/mol)**
Poly(butadiene), $PB_{1,4}$	1,900 – 3,900<br>cis: ~3,000	2,600	5,900
Polystyrene, PS	13,300 – 18,700	16,500	31,200
Poly(dimethylsiloxane), PDMS	6,650 – 12,000	10,000	24,500

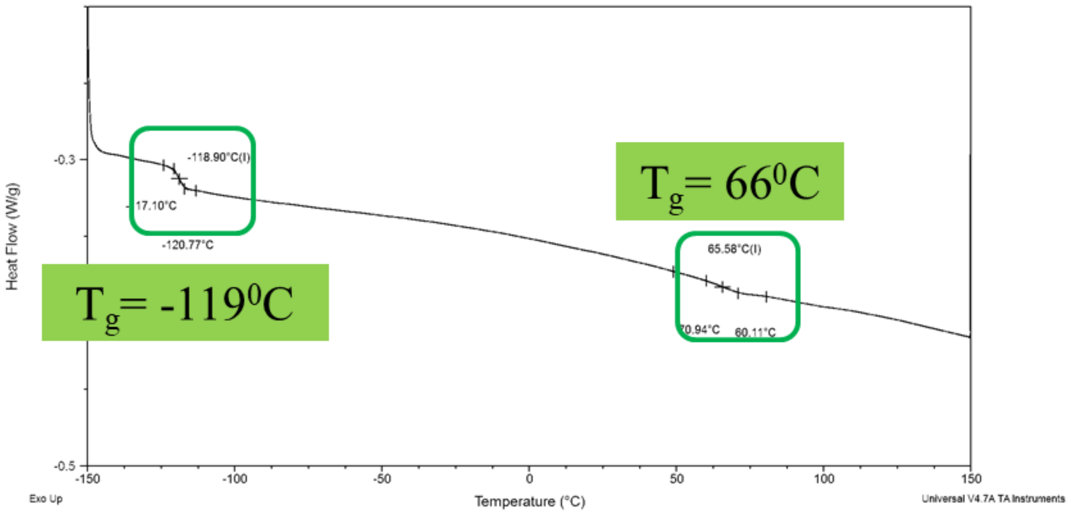

Figure S7. DSC thermograph of the PS-*b*-$PB_{1,4}$-*b*-PDMS triblock terpolymer (sample 1), where only the two T_gs of PS and PDMS are evident.

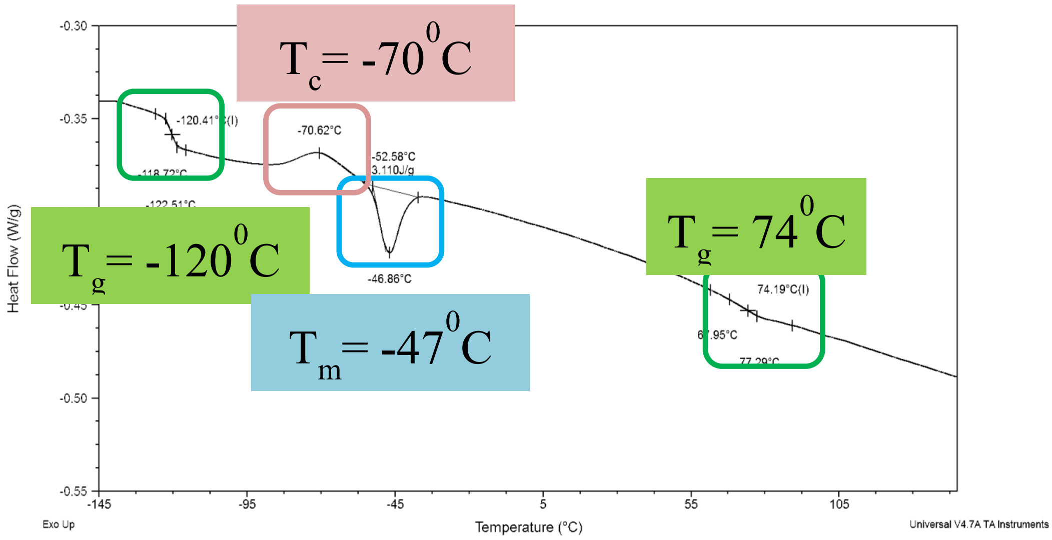

Figure S8. DSC thermograph of the PS-*b*-PB1,4-*b*-PDMS triblock terpolymer (sample 2), where only the two T_gs of PS and PDMS are evident together with the T_c and T_m of PDMS.

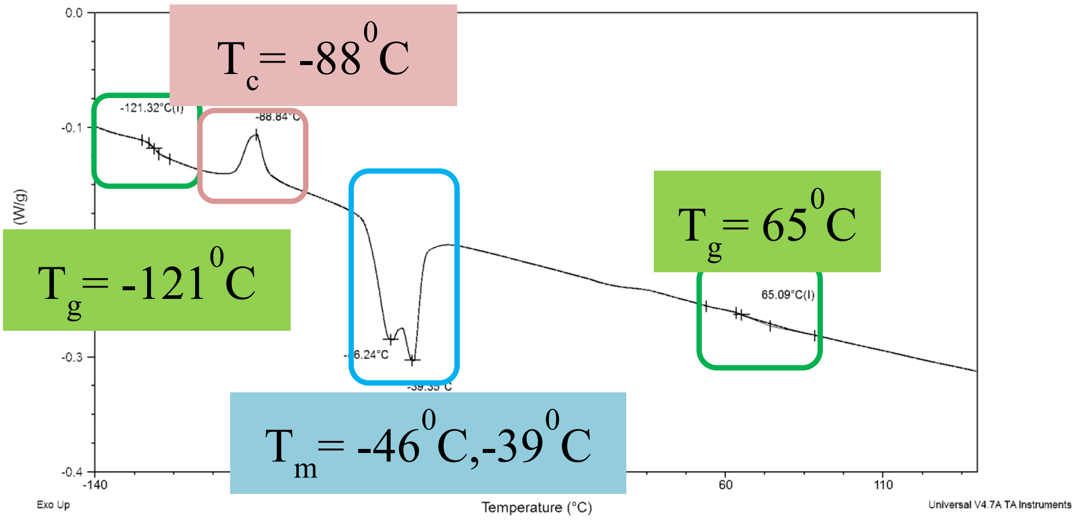

Figure S9. DSC thermograph of the PB1,4-*b*-PS-*b*-PDMS triblock terpolymer (sample 3), where only the two T_gs of PS and PDMS are evident together with the T_c and T_m of PDMS.

D). Estimation of the Flory–Huggins Interaction Parameter (χ)

The χ_{ij} parameter can be estimated from the solubility parameters of a pair of polymer chains *i* and *j* as follows [4-7]:

$$\chi_{ij} = \frac{V}{k_B T}\left(\delta_i - \delta_j\right)^2 \qquad (1)$$

$$E_{coh} = \delta^2 V \qquad (2)$$

$$V = \sqrt{V_1 V_2} \qquad (3)$$

$$V_{1,2} = MB_{1,2}/d_{1,2}N_A \quad (4)$$

where, χ_{ij} is the Flory–Huggins interaction parameter between segments i and j, k_B is the Boltzmann constant, δ_i and δ_j are the solubility parameters of chains i and j respectively, T (K) is the annealing temperature of preparation of the sample for TEM and SAXS, V is the geometric average of the molecular volume of the monomeric unit of each type of i (or j) and E_{coh} is the coherent energy of the corresponding monomeric unit. The E_{coh} and V values of the monomeric units for the three different blocks were calculated based on the ligand contribution of the monomeric units according to Fedors theoretical values.[6] The resulting χ_{ij} for the different segments i and j as calculated from Equations (1-4) are exhibited in Table 2. In Table S3, the interaction parameter for known segment-pairs is also given.

Table S3. Flory-Huggins χ interaction parameter for PS/PDMS and PS/$PB_{1,4}$

χ	**PS**	**1,4-PB**	**PDMS**
**PS**	-	0.065	0.479
**1,4-PB**	0.065	-	0.142

References

1. www.polymerdatabase.com
2. Fetters, L. J.; Lohse, D. J.; Richter, D.; Witten, T. A.; Zirkel, A. Connection between Polymer Molecular Weight, Density, Chain Dimensions, and Melt Viscoelastic Properties. *Macromolecules* **1994**, *27*, 4639-4647.
3. Ferry, D. J. In *Viscoelastic Properties of Polymers*, 3rd ed.; John Wiley & Sons Inc: New York, **1980**.
4. Politakos, N.; Ntoukas, E.; Avgeropoulos, A.; Krikorian, V.; Pate, B. D.; Thomas, E. L.; Hill, R. M. Strongly Segregated Cubic Microdomain Morphology Consistent with the Double Gyroid Phase in High Molecular Weight Diblock Copolymers of Polystyrene and Poly(dimethylsiloxane). *J. Polym. Sci: Part B: Polym Phys*. **2009**, *47*, 2419-2427.
5. Almdal, K.; Hillmyer, M. A.; Bates, F. S. Influence of Conformational Asymmetry on Polymer-Polymer Interactions: An Entropic or Enthalpic Effect? *Macromolecules* **2002**, *35*, 7685-7691.
6. Miller-Chou, B. A.; Koenig, J. L. A review of polymer dissolution. *Prog. Polym. Sci.* **2003**, *28*, 1223-1270.
7. Avgeropoulos, A.; Rangou, S.; Krikorian, V.; Thomas, E. L. Synthesis and Self-Assembly of 2nd Generation Dendritic Homopolymers and Copolymers of Polydienes with Different Isomeric Microstructures. *Macromol. Symp.* **2008**, *267*, 16-20.
